# Fabrication of a Horizontal and a Vertical Large Surface Area Nanogap Electrochemical Sensor

**DOI:** 10.3390/s16122128

**Published:** 2016-12-14

**Authors:** Jules L. Hammond, Mark C. Rosamond, Siva Sivaraya, Frank Marken, Pedro Estrela

**Affiliations:** 1 Department of Electronic & Electrical Engineering, University of Bath, Bath BA2 7AY, UK; j.l.hammond@bath.ac.uk (J.L.H.); s.sivaraya@bath.ac.uk (S.S.); 2 School of Electronic & Electrical Engineering, University of Leeds, Leeds LS2 9JT, UK; m.c.rosamond@leeds.ac.uk; 3 Department of Chemistry, University of Bath, Bath BA2 7AY, UK; f.marken@bath.ac.uk

**Keywords:** nanogap, horizontal coplanar, vertical coplanar, anodic bonding, dielectric, capacitance

## Abstract

Nanogap sensors have a wide range of applications as they can provide accurate direct detection of biomolecules through impedimetric or amperometric signals. Signal response from nanogap sensors is dependent on both the electrode spacing and surface area. However, creating large surface area nanogap sensors presents several challenges during fabrication. We show two different approaches to achieve both horizontal and vertical coplanar nanogap geometries. In the first method we use electron-beam lithography (EBL) to pattern an 11 mm long serpentine nanogap (215 nm) between two electrodes. For the second method we use inductively-coupled plasma (ICP) reactive ion etching (RIE) to create a channel in a silicon substrate, optically pattern a buried 1.0 mm × 1.5 mm electrode before anodically bonding a second identical electrode, patterned on glass, directly above. The devices have a wide range of applicability in different sensing techniques with the large area nanogaps presenting advantages over other devices of the same family. As a case study we explore the detection of peptide nucleic acid (PNA)−DNA binding events using dielectric spectroscopy with the horizontal coplanar device.

## 1. Introduction

There is increasing motivation to develop low-cost parallel assays for point-of-care devices for disease diagnostics and environmental monitoring. Direct electrical detection with impedimetric, amperometric, and capacitance/dielectric spectroscopy measurements offer greater suitability for monolithic chip integration with signal processing circuitry. These direct electrical techniques do not require an expensive and time-consuming labelling step, making them more amenable to large scale manufacturing.

Sensors comprised of electrodes with interelectrode spacing on the order of nanometers are termed 'nanogap' sensors. By using electrodes separated by a nanogap, very large electric fields (>1 MV·m^−1^) can be established using fairly modest applied potentials (<50 mV). Nanogap sensors, therefore, provide a highly sensitive platform for the detection of biomolecules whilst minimising the sample volume and allow a wide range of electrical behaviours to be observed. 

Early forms of nanogaps were predominantly horizontal coplanar devices and a range of fabrication techniques exist, including: electron-beam lithography (EBL) [1,2], mechanical break junctions [3], focused ion beam (FIB) milling [4,5], oxidative plasma ablation [6], electromigration [7,8], electroplating [9], molecular rulers [10], chemical-mechanical polishing (CMP) [11] electrochemical synthesis [12], direct chemical synthesis [13], and dip-pen nanolithography (DPN) [14].

Originally, vertical coplanar nanogaps predominantly existed in the guise of scanning electrochemical microscopes (SECMs) [15]. However, in more recent years, new methods to fabricate vertical coplanar nanogaps have emerged, including: nanoskiving [16], etching recesses into sidewalls [17], etching sacrificial layers [18,19,20,21], and molecular-beam epitaxy [22]. 

There are two distinct types of nanogap devices that have emerged. The first is those that are used for the interrogation of biomolecules. These often consist of triangular point-like electrodes with minute interelectrode distances to match that of the target molecule and minimal contact area for improved selectivity. The second are those used for electrochemical sensing applications, where both reduced interelectrode distance and large surface area can lead to improved performance. 

Two electrochemical techniques of special interest are redox cycling and dielectric spectroscopy. Redox cycling involves polarising two closely-spaced electrodes so that an analyte can be repeatedly cycled between a reduced and oxidised state. This leads to a single molecule contributing to the current response on each reaction, effectively amplifying the sensor response. Here, the response depends critically on minimising the interelectrode spacing to reduce the time for interdiffusion, as well as maximising the available surface area for the electrochemical reactions. Dielectric spectroscopy is gaining importance as a label-free detection tool for monitoring biomolecular binding events. Here larger surface areas allow increased immobilisation of the recognition probe. In turn, reducing the interelectrode spacing means that the electric double layers occupy an increased fraction of the sample volume, mitigating screening effects and increasing sensitivity.

For the interested reader there are several comprehensive reviews [23,24,25] covering the fabrication and use of nanogap sensors. Either way, there is still further research to be done to reduce fabrication costs and improve the feasibility of large-scale production of nanogap devices. The majority of longer horizontal nanogap devices are fabricated using either EBL or FIB. Vertical nanogap devices for electrochemical sensing applications have, in particular, been pioneered by the groups of both Lemay [19,20,26] and Wolfrum [19,20,27,28,29]. By far the most popular method for the fabrication of vertical nanogap devices involves etching away a sacrificial layer between two electrode layers. However, complete removal of this layer becomes extremely difficult for large areas without causing collapse. 

Table 1 shows a range of both horizontal and vertical nanogap devices suited for electrochemical sensing. The listed devices have been fabricated using a variety of methods and have gap sizes ranging from ~50 nm to ~510 nm and electrode surface areas ranging from ~1.6 × 10^−13^ m^2^ to ~3.0 × 10^−6^ m^2^.

In this work we present two different approaches for creating large-area nanogap sensors. The devices feature ideal parallel plate geometry in both vertical and horizontal orientations. Both techniques could be adopted for mass-production. The first device uses EBL to create an 11 mm long, ~215 nm serpentine nanogap with an integrated microfluidic layer for sample delivery. The serpentine design maximises the length in the writeable EBL area whilst maintaining a single nanogap. It is envisaged that the EBL processing could, in the future, be replaced with wafer-level nanoimprint lithography (NIL) to improve throughput and reduce cost. The second method uses very simple optical lithography and a dry anisotropic etch to form a well-controlled sub-micron depth channel. This channel allows a large lower electrode to be buried before an identical upper electrode patterned on glass is anodically bonded directly above, forming a ~500 nm nanogap. 

## 2. Fabrication of Horizontal and Vertical Coplanar Nanogap Sensors 

All optical lithography was performed using 4” chrome masks (CBL4009Du-AZ1500) patterned using a µPG 101 direct laser writer (DLW) (both from Heidelberg Instruments Mikrotechnik GmbH, Heidelberg, Germany) by standard contact microlithography. Photomask designs were created using CleWin4 layout editor (WieWeb software, Hengelo, Netherlands). SEM images were taken using a 1530 VP FESEM (LEO Elektronenmikroskopie GmbH, Oberkochen, Germany). Layer thicknesses were determined using a Dektak^®^ 8 mechanical profilometer (Veeco Instruments Inc., Plainview, NY, USA). Spincoating, baking, exposure, and development conditions were as per the manufacturers’ guidelines unless otherwise stated. Fabrication was performed at the die level with the dies incorporating a large, empty perimeter to aid sample handling.

### 2.1. Horizontal Coplanar Nanogap Device

#### 2.1.1. Device Details

**Figure 1:**
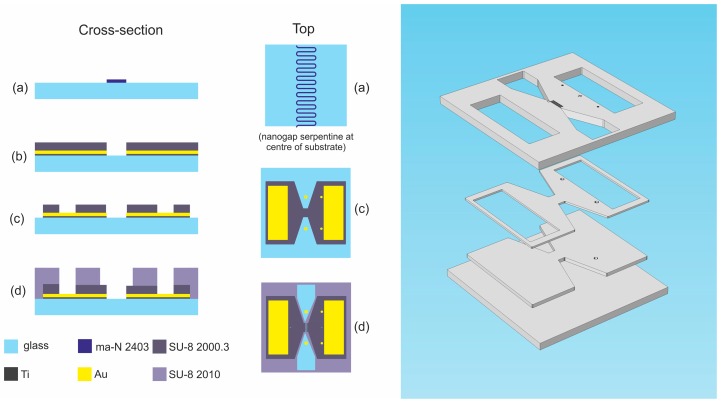
**Left:** Diagram showing the cross-section and top views of device after: (**a**) patterning of ma-N 2403 resist for electrode liftoff; (**b**) etching of SU-8 in nanogap; (**c**) etching to define device area and provide access to contact pads; and (**d**) patterning of microfluidic serpentine. **Right:** Exploded 3D diagram of device layers.

The device is comprised of a central ~200 nm nanogap consisting of twenty 400 µm straight sections connected with twenty arcs of 50 µm radius to form an 11 mm long serpentine, maximising the length of the nanogap in the writeable area of the electron-beam lithography (EBL) system. The electrodes either side of the nanogap are addressed by large 4 mm × 12 mm rectangular contact pads. On top of the nanogap sits a 35 µm wide microfluidic serpentine with funnelled inlets and outlets to ensure a laminar flow regime for a wide range of flow rates (0.5 nL·min^−1^ to 10.0 µL·min^−1^). Figure 1 shows the cross-sectional and top views at different stages of the fabrication process, as well as a 3D exploded view of the key layers.

#### 2.1.2. Fabrication Procedure

Electrode layer: First a 3” BOROFLOAT^®^ 33 glass wafer (PI-KEM Ltd., Tamworth, UK) was diced into 20 mm × 20 mm dies and cleaned in acetone, then isopropyl alcohol (IPA), with ultrasonic agitation. The cleaned glass dies were then dehydrated on a hotplate at 200 °C for 10 min and allowed to cool. Next a 300 nm ma-N 2403 negative tone resist (MicroChem Corp., Westborough, MA, USA) was spun at 4000 rpm for 40 s. A 5 nm Al layer was then thermally evaporated to serve as an anti-charging layer. The ma-N 2403 resist was patterned to form an 11 mm long, 200 nm wide serpentine using a JBX-6300FS EBL instrument (JEOL Ltd., Akishima, Japan) (exposure conditions: 100 kV, 3.0 nA beam current, 4 nm shot pitch, 1600 µC·cm^−2^ dose) and BEAMER software (GenISys GmbH, Munich, Germany) was used to optimise the dose. The Al layer was then etched in PAN etchant (H_3_PO_4_:HNO_3_:HAc:H_2_O, 80:5:5:10, vol.) with manual agitation for 90 s and the resist developed in MICROPOSIT^™^ MF-322 (micro resist technology GmbH, Berlin, Germany). The electrode layer was formed by an electron-beam evaporation of a 5 nm Ti adhesion layer followed by 150 nm Au (multi-step deposition). Liftoff was performed in *N*-Methyl-2-pyrrolidone (NMP) (MICROPOSIT^™^ Remover 1165, micro resist technology GmbH) at 80 °C for 10 min followed by 5 min with 50% ultrasonic agitation. 

**Figure 2:**
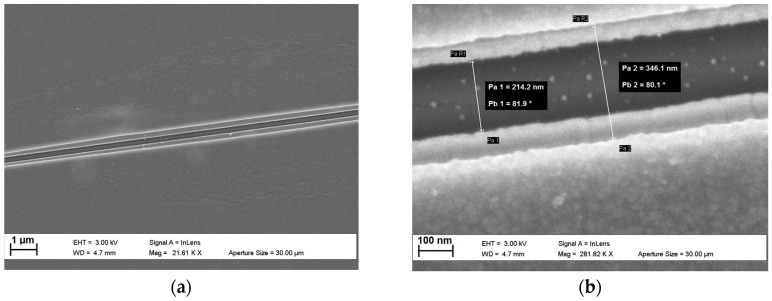
(**a**) SEM image showing a 13 µm section with both the gold electrodes and SU-8 passivation layer visible; and (**b**) measurements of the electrode and passivation layer nanogaps.

Passivation layer: A diluted SU-8 2002 (MicroChem Corp.) layer (8 g SU-8 2002 : 15 g cyclopentanone) was flood-exposed (10 s at 14.6 mW·cm^−2^ (365 nm)) using an EVG^®^ 610 semi-automated mask alignment system, (EV Group, St. Florian am Inn, Austria); this forms the passivation layer on top of the electrodes. To pattern this layer a second ma-N2403 liftoff layer aligned to the initial layer was exposed using EBL (exposure conditions: 100 kV, 3.0 nA beam current, 4 nm shot pitch, 1600 µC·cm^−2^) and developed in MICROPOSIT^™^ MF-322 (micro resist technology GmbH). A 10 nm Al layer was then thermally evaporated before liftoff in NMP. Using the Al as a hard mask the SU-8 in the nanogap was removed with a reactive-ion-etching (RIE) step using a low-pressure oxygen chemistry (150 W, 4 min) before removing the Al layer using PAN etchant. Figure 2 shows a straight 13 µm section of the passivated nanogap. The nanogap was measured to be around 214 nm, with the thin SU-8 passivation layer receded 60 nm perpendicular from each electrode face.

**Figure 3:**
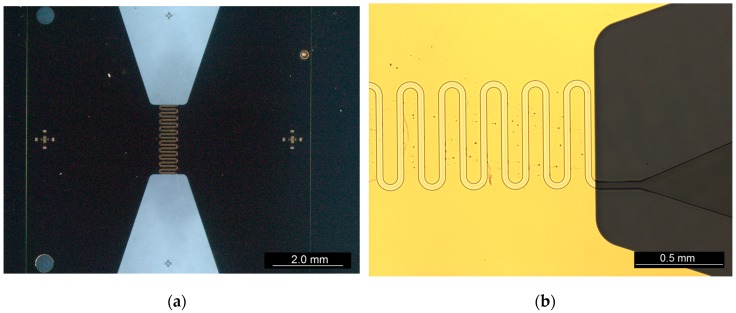
(**a**) The bow-tie-shaped sensor with central microfluidic channel; and (**b**) close-up showing the serpentine microfluidic channel with funnelled inlet/outlet.

Contact pads and microfluidic layer: With the passivated nanogap now formed, a MICROPOSIT^™^ S1813 (micro resist technology GmbH) layer was optically patterned (3.2 s at 14.6 mW·cm^−2^ (365 nm), EVG^®^ 610) and developed in MICROPOSIT^™^ MF-319 (micro resist technology GmbH). This layer was used as an etch mask to define a bow-tie-shaped electrode area using a KI:I_2_:H_2_O (8:2:80, vol.) wet etch to remove the Au and a H_2_O:HF (10:1, vol.) wet etch to remove the underlying Ti. The S1813 etch mask was then removed in acetone before optically patterning (3.2 s at 14.6 mW·cm^−2^ (365 nm), EVG^®^ 610) a second S1813 etch mask. Access to rectangular Au contact pads was provided using a second low-pressure O_2_ RIE process (150 W, 1 min 55 s). This second S1813 layer was then again removed in acetone before optically patterning (EVG^®^ 610) a 10 µm SU-8 2010 (MicroChem Corp.) microfluidic layer with a 35 µm wide serpentine using a dose of 1400 mJ·cm^−2^ with a ZJB360 long-pass (λ_c_ = 365 nm) optical filter (Omega Optical Inc., Brattleboro, VT, USA) to reduce 'T-topping' before developing with MICROPOSIT**^™^** EC solvent (micro resist technology GmbH) and hardbaking at 150 °C for 20 min. The completed device is shown in Figure 3. 

Based on the 20 mm × 20 mm die, 6/12 dies per wafer could be produced on 3”/4” wafers, respectively. After optimising the fabrication procedure yield was 100% (five devices). Throughput of the devices could be significantly improved by adopting the use of wafer-level nanoimprint lithography (NIL) with a silicon ‘master’ patterned by EBL, as well the use of a self-aligned passivation layer instead of the thin SU-8 layer.

#### 2.1.3. I-V Response

**Figure 4:**
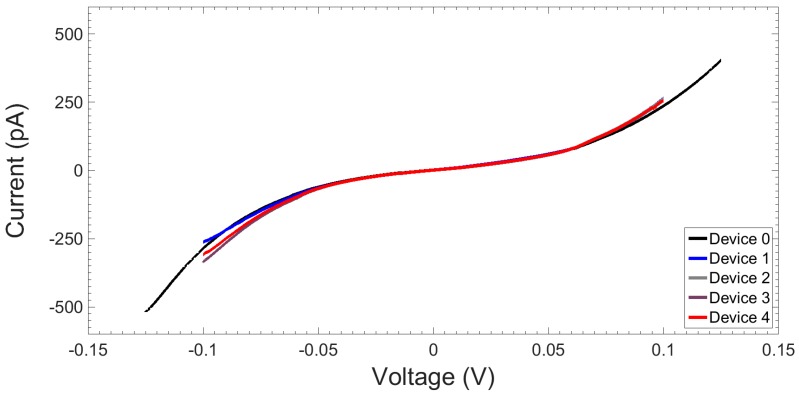
Measured I-V curve showing leakage currents in air for the horizontal coplanar (~215 nm) nanogap test device (Device 0) and the four devices (Devices 1–4) used in later PNA−DNA binding experiments.

I-V characteristics in air for one test device and the four devices used in the later peptide nucleic acid (PNA)−DNA experiments were measured using a B1500A semiconductor device analyser (Agilent Technologies Inc., Santa Clara, CA, USA) and a probe station (Wentworth Laboratories Inc., Brookfield, CT, USA) placed on an isolation table (Newport Spectra-Physics Ltd., Oxford, UK) equipped with four micropositioners (JMicron Technology Corp., Hsinchu, Taiwan), all housed in a large Faraday cage. Measurements were taken using a four-terminal configuration. Figure 4 shows that the leakage current at −125 mV is ~500 pA, equating to an isolation of ~0.25 GΩ. Increasing the potential window further eventually led to breakdown of the devices. However, it was found that the device could be recovered by permitting higher currents in order to ‘blow’ the shorts. After recovering from breakdown the leakage current increased up to ~1.5 nA at 100 mV, equating to an isolation of ~67 MΩ.

### 2.2. Vertical Coplanar Nanogap Device

#### 2.2.1. Device Details

**Figure 5:**
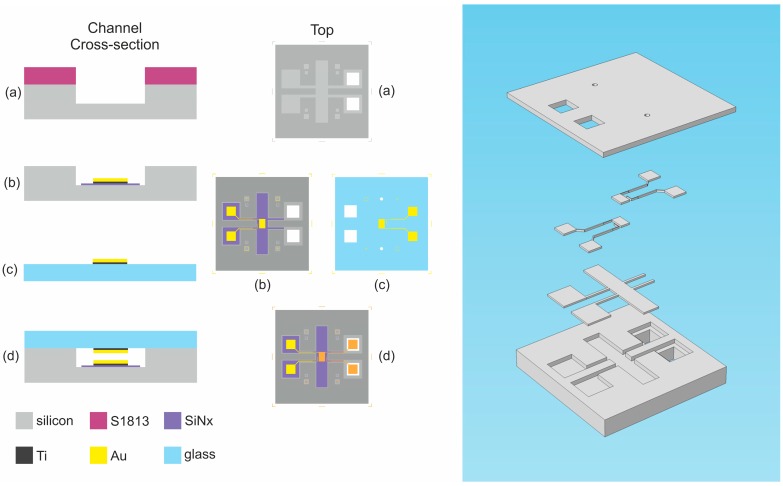
**Left:** Sequence diagram showing the channel central cross-section and top views of device after: (**a**) anisotropic dry etching of silicon to create a channel using a S1813 mask; (**b**) patterning of silicon nitride (SiN_x_) passivation layer and lower electrode on silicon; (**c**) patterning the upper electrode on glass; and (**d**) anodic bonding. **Right:** Exploded 3D diagram showing the different layers of the completed device.

The second device uses an anisotropic dry etch process to create a 2.0 mm × 10.0 mm central channel in silicon with a sub-micron depth. At the centre of this channel sit two large 1.0 mm × 1.5 mm sensing electrodes and two small 40 µm × 800 µm auxiliary electrodes. The generous width of the channel with respect to the electrodes provides improved tolerance to misalignment, as well as reducing the likelihood of channel collapse during the bonding process. The electrodes are patterned on two substrates, silicon and glass, and then anodically bonded together to create a 500 nm vertical coplanar nanogap separation. Wires connect these four electrodes to electrical contact pads at both sides of the device, and access to these contact pads are provided with laser micromachined apertures in both the silicon and glass. Laser micromachining offers high accuracy, repeatability, reproducibility, and low surface roughness with medium throughput capability [36]. Figure 5 depicts the key steps in the fabrication sequence along with a 3D exploded view of the completed device.

#### 2.2.2. Fabrication Procedure

Substrate preparation: 3” silicon wafers (p-type, <100>, 380 ± 50 µm, 1–10 Ω·cm SSP) and 3” BOROFLOAT^®^ 33 glass wafer (both from PI-KEM Ltd.) were diced into 15 mm × 15 mm dies. 

Access to electrical contact pads was provided by laser micromachining 2 mm × 2 mm square apertures in both the silicon and glass dies. Two 500 µm diameter holes were also laser micromachined in the glass to create microfluidic inlets and outlets. All of the substrates were cleaned in acetone and then IPA, both with aggressive ultrasonic agitation, before finally cleaning in Piranha solution (H_2_SO_4_:H_2_O_2_, 3:1, vol.) for 10 min followed by a DI water rinse and N_2_ dry. 

Channel formation: An S1813 etch mask was optically patterned using standard contact microlithography (150 mJ·cm^−2^) (Karl Süss MJB3, Süss Microtec AG, Garching bei München, Germany) and developed in MICROPOSIT^™^ 351 (micro resist technology GmbH). Using a SF_6_/O_2_ chemistry the silicon was etched using inductively-coupled plasma (ICP) reactive ion etching (RIE) (Plasmalab System 100, Oxford Instruments plc, Abingdon, UK), as per the recipe in Table 2, to produce a 900 nm deep channel. The depth of the channel was found to be a linear function of the etch duration, providing a simple way of tuning the electrode separation. Improved control could be achieved by reducing the RF power. The etch mask was then removed in acetone. To isolate the lower electrode first, the native oxide was removed using a buffered oxide etch (BOE) (5:1) for 3 min, followed by a DI water rinse and N_2_ dry. Then a 110 nm silicon nitride (SiN_x_) layer was blanket deposited by multi-step plasma-enhanced chemical vapour deposition (PECVD) (Plasma-Therm 790, Plasma-Therm, Saint Petersburg, FL, USA) using a SiH_4_/NH_3_ chemistry at 300 °C (250 SCCM SiH_4_, 2.5 SCCM NH_3_, 50 W RF). This layer was patterned using a BOE etch (5:1) for 5 min with an optically-patterned S1813 etch mask. 

Electrode layers: The same photomask was used for both the lower (on SiN_x_) and upper electrodes (on glass) to expose a 2 µm AZ^®^ nLOF 2020 (MicroChemicals GmbH, Ulm, Germany) liftoff mask, followed by electron-beam evaporation of a 5 nm Ti adhesion layer and 140 nm Au layer (layer thicknesses determined by quartz crystal microbalance (QCM) monitor). Liftoff was performed in MICROPOSIT^™^ Remover 1165 (micro resist technology GmbH) at 65 °C for 15 min with 50% ultrasonic agitation.

Bonding: To finalise the sensor fabrication, the glass and silicon substrates were anodically bonded at 225 V, 250 °C for 2.5 h to form an interelectrode separation of 500 nm. This process required careful optimisation to prevent channel collapse and is discussed in more detail below.

**Figure 6:**
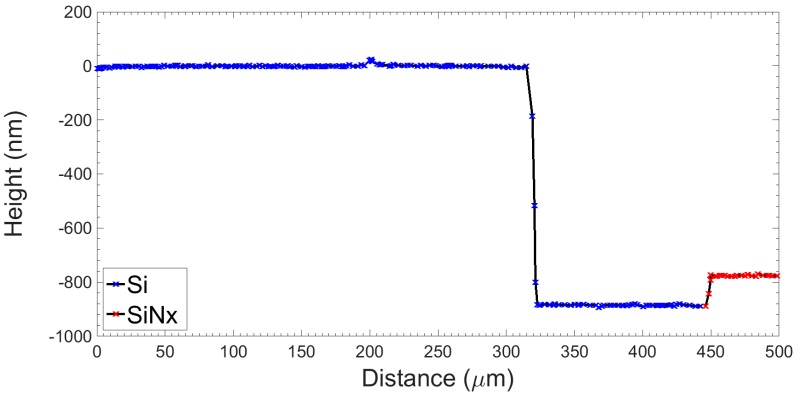
Vertical profile showing a 900 nm silicon channel and a 110 nm silicon nitride (SiN_x_) passivation layer prior to electrode deposition and anodic bonding.

A representative vertical profile of the channel and passivation layer prior to electrode deposition and anodic bonding is shown in Figure 6. The depth of the silicon channel was measured as 900.5 nm ± 3.5 nm (n = 20, at five die positions, four separate dies). The silicon nitride layer was measured as 110.4 nm ± 2.3 nm (n = 10, at five die positions, two separate dies).

#### 2.2.3. Anodic Bonding

By applying a sufficient DC voltage between the silicon and glass, positive alkali ions in the glass (Na^+^ and K^+^) are displaced to form a depletion layer at the interface. Oxygen anions drifting towards the silicon lead to the formation of an oxide layer which in turn contributes to the migration of the bonding front [37]. The resultant bond is often much stronger than the glass or silicon itself, with measured values varying between 5 and 25 MPa [38]. Elevated temperatures improve ion mobility and a higher voltage increases the electric field, both of which reduce the required bonding time, however, these must be carefully selected to prevent collapse of the relatively wide channels. 

To improve the efficiency of the anodic bonding process, <100> p-type silicon and high Na_2_O/K_2_O content (4%) borosilicate glass (BOROFLOAT^®^ 33, PI-KEM Ltd.) wafers were chosen for the substrates based on the work by Lee et al. [37]. The two halves of the device were cleaned with acetone, then IPA, dried with N_2_, and brought into intimate contact with the electrodes facing each other. Alignment was performed under a stereoscope and then a small force was applied using a jig with a clear Perspex^®^ plate to form a prebond. The design incorporated plenty of alignment tolerance to prevent fouling in the case of misalignment. 

The prebonded device was placed between two steel plates held together with alumina bolts and placed inside a tube furnace (Carbolite Ltd., Hope Valley, UK) at 250 °C. Two wires extending from the furnace to electrically connect the steel plates were insulated with ceramic and connected to a N5751 high-voltage power supply (Agilent Technologies Inc.). A voltage of 225 V was applied between the steel plates (glass held at 0.0 V) with the supply current limited to 14 mA. Bonding current was monitored using a data-logging U1282A digital multimeter (Keysight Technologies Inc., Santa Rosa, CA, USA). 

**Figure 7:**
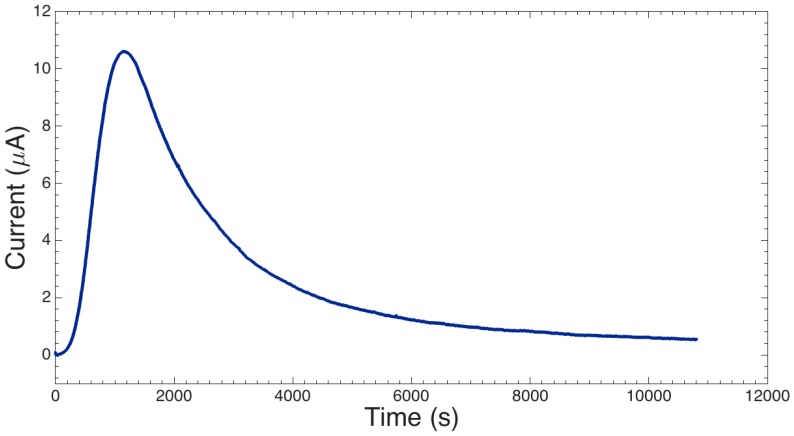
Plot showing the anodic bonding current over an extended period of 3 h (10,800 s). Bonding conditions were 225 V at 250 °C.

Figure 7 shows the typical current response during anodic bonding. Initially the current rises as the intimately-contacted area increases until a maximum is reached; bonding then continues until a plateau is reached (around 10% of peak current). Bonding could be easily paused and restarted without any consequence. With the selected voltage and temperature, bond time was approximately 2.5 h. Peak current was ~11 μA and with a total bond area of 100.5 mm^2^, the peak current density is around 0.1 μA·mm^−2^. Experiments showed that devices with greater intimately contacted areas formed during the prebonding stage led to higher initial currents, in agreement with the model developed by He et al. [39]. Increasing the voltage or temperature led to decreased yield. At 300 V, 300 °C all devices (8/8) exhibited partial or total channel collapse. At the optimised conditions of 225 V and 250 °C the yield after anodic bonding was 7/7. With a die size of 15 mm × 15 mm, a total of 12/22 dies per wafer can be produced on 3”/4” wafers, respectively. Given that the fabrication steps prior to bonding are fairly standard cleanroom processes, we believe that this technique could provide a relatively high throughput of devices. However, manually aligning the individual 15 mm × 15 mm dies prior to prebonding naturally leads to some misalignment. The generous tolerances in the design mitigate fouling but will affect the sensor characteristics between devices. It is anticipated that aligning at the wafer level with a purpose-made apparatus would reduce these errors. 

**Figure 8:**
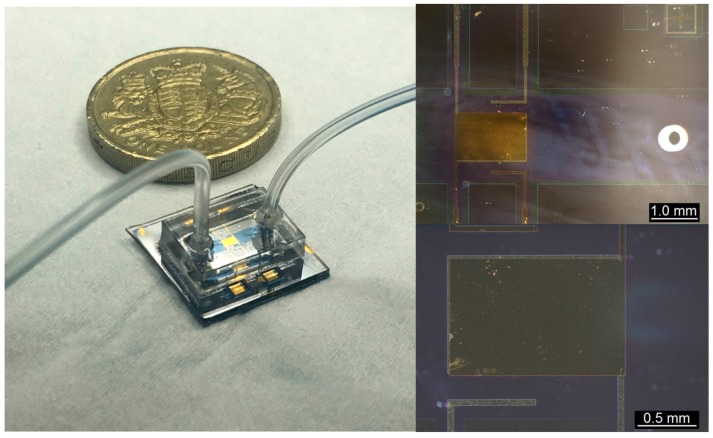
**Left:** Photograph showing a completed device with PMMA microfluidic interface block, pound coin for scale. **Top right:** Microscope image showing the overlapping sensing electrodes and auxiliary electrodes with a laser micromachined microfluidic hole centred vertically in the channel. **Bottom right:** Close-up microscope image showing misalignment of the two central electrodes.

A completed device is shown in Figure 8. Contact to the lower electrodes is made through the square apertures in the glass with micropositioners. The upper electrodes are electrically connected through the square apertures in the silicon to PCB landing pads using a small length of protruding Kynar™ wire and Ag conductive paste. These landing pads are then wired to larger contact pads that can be addressed using micropositioners. Finally a poly(methyl methacrylate) (PMMA) block is attached to the glass to provide connections to microfluidic tubing. 

#### 2.2.4. Electrical Characterisation

**Figure 9:**
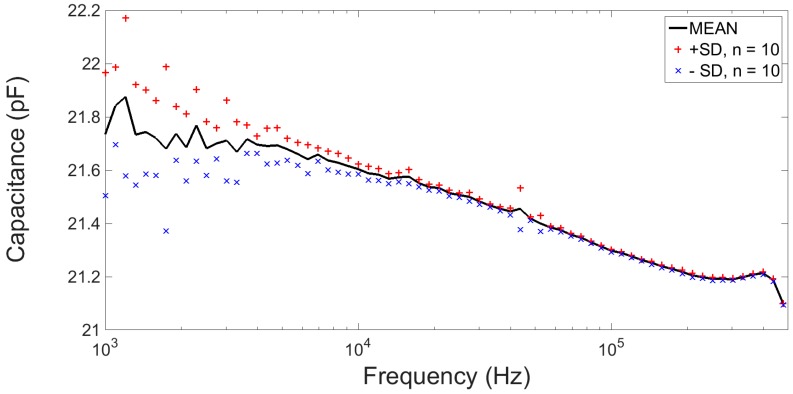
Capacitance profile for a 500 nm vertical nanogap device filled with air for the frequency range 1 kHz to 500 kHz.

The capacitance response of a vertical nanogap device is shown in Figure 9. The measurement setup consisted of a B1500A semiconductor device analyser fitted with a 1 kHz to 5 MHz multi-frequency capacitance measurement unit (MFCMU) (Agilent Technologies Inc.). Connections to the device were made using micropositioners (JMicron Technology Corp.) with a shielded two-terminal configuration using a probe station (Wentworth Laboratories Inc.) on an isolation table (Newport Spectra-Physics Ltd.), all housed in a Faraday cage. The response was recorded for a frequency range of 1 kHz to 500 kHz with a 0.0 V DC bias and 10 mV AC signal using a 'long' integration time (16 power line cycles). 

I-V sweeps were also performed using the same setup described in Section 2.1.3 except measurements were performed with a two-terminal configuration due to the backside contact. Applying a voltage sweep of ± 500 mV, the current response was at the level of the measurement noise, <1 pA, indicating an isolation >0.5 TΩ.

## 3. Dielectric Spectroscopy Sensing of DNA

In order to demonstrate a direct electrical detection application using a nanogap sensor, the horizontal coplanar nanogap device described in Section 2.1 was used to perform dielectric spectroscopy sensing of single-stranded (ss) DNA hybridisation with a ssPNA probe layer in phosphate-buffered saline (PBS). PBS was selected as it has an osmolarity and ion concentration similar to those of the human body. The ssPNA probe layer was used as the ssPNA structure does not carry a negative charge at physiological pH and this leads to stronger binding with ssDNA due to the lack of electrostatic repulsion [40]. Two devices were used to test the response to complementary ssDNA and two other devices were used as controls with complete mismatch ssDNA sequences.

### 3.1. Detection Scheme

The double-layer capacitance formed by the accumulation of counter-ions near the electrode surface is sensitive to the changes in dielectric and charge environment at the electrode−electrolyte interface. Binding of the ssDNA to the ssPNA probe layer leads to a conformational change that interferes with the electric double layer and charge transport between the polarised electrodes. Detection is based on changes in the measured sensor capacitance over a wide frequency range as a result of the formation of the PNA−DNA duplex.

### 3.2. Experimental Details

#### 3.2.1. Reagents

Dimethyl sulfoxide (DMSO), ethylenediaminetetraacetic acid (EDTA), 6-mercapto-1-hexanol (MCH), and phosphate-buffered saline (PBS) tablets (10 mM, pH 7.4 at 25 °C) were all purchased from Sigma-Aldrich Ltd., Dorset, UK. All aqueous solutions were prepared using 18.2 MΩ·cm ultra-pure class 1 DI water treated with a Biopak^®^ polishing pack (Merck Millipore, Darmstadt, Germany).

Thiolated ssPNA (Cambridge Research Biochemicals, Billingham, UK) with sequence HS-C6-AEEEA-ACA-ACA-ACA-ACA-ACA (N- to C-terminus, where AEEEA is a 9-amino-4,7-dioxanonanoic acid linker) was suspended in a 1:1 volumetric ratio of DMSO:DI to create a 100 µM stock. This stock was heated to 55 °C for 10 min in a dry block heater followed by vortex (30 s) then ultrasonication (1 min) before diluting to 1 µM aliquots in DMSO:DI (1:1, vol.) for immobilisation.

Complementary TGT-TGT-TGT-TGT-TGT and complete mismatch CAC-CAC-CAC-CAC-CAC ssDNA sequences (both from Sigma-Aldrich Ltd.) were suspended in a 10 mM Tris-HCl pH 7.4, 1 mM buffer to form a 100 µM stock before preparing 10 µL aliquots of working concentrations in PBS.

#### 3.2.2. Probe Layer Fabrication

The sensors were first cleaned by rinsing with acetone, IPA then DI water and drying with N_2_. The ssPNA probe layer surface density was controlled by the co-immobilisation with MCH (1 µM:4 µM) in DMSO:DI (1:1, vol.). The sensors were incubated with 10 µL of solution, placed in a humidity chamber at 5 °C overnight for 16 h. To ensure complete thiol coverage of the gold surface, the sensors were first rinsed with DI water then backfilled with 10 µL of 1 mM MCH (in DMSO:DI, 1:1, vol.) for 1 h at room temperature then rinsed and stabilised with PBS for 1 h before measurements.

#### 3.2.3. Dielectric Spectroscopy Measurements

Measurements were taken of the stabilised ssPNA probe layer then with increasing concentrations of the complementary and complete mismatch non-complementary (control) ssDNA sequences. Between measurement steps the sensors were rinsed with PBS, dried with N_2_, and then incubated for 30 min at room temperature with 5 µL of ssDNA sequences. The sensors were then washed in PBS, dried with N_2_, incubated with PBS, and stabilised for five minutes before taking measurements.

The measurement setup consisted of a B1500A semiconductor device analyser fitted with a 1 kHz to 5 MHz multi-frequency capacitance measurement unit (MFCMU) (Agilent Technologies Inc.). Connections to the sensor were made using micropositioners (JMicron Technology Corp.) with a four-terminal pair configuration using a probe station (Wentworth Laboratories Inc.) on an isolation table (Newport Spectra-Physics Ltd.), all housed in a Faraday cage. Open, short, and phase corrections were performed prior to measurements to correct for residual admittance, impedance, and phase in the system, respectively. The capacitance was recorded between 1 kHz and 1 MHz with a 0.0 V DC bias and a 10 mV AC signal with a ‘long’ integration time (16 power line cycles). In this work we report the parallel capacitance (C_p_), automatically calculated from the measured susceptance. Five scans were taken and averaged for each device at each step, with the probe needles repositioned after each scan. All measurements were taken on the same day with temperature varying from 20.4 to 22.5 °C, and relative humidity from 58%–64%.

### 3.3. Results

**Figure 10:**
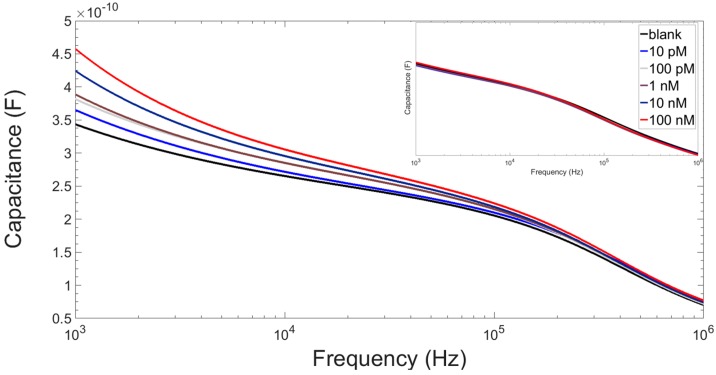
Capacitance profile for a device with a ssPNA probe layer after hybridisation with increasing concentrations of complementary ssDNA. **Inset:** Capacitance profile for the control device response to complete mismatch non-complementary ssDNA.

Figure 10 shows the dose response of a test device for increasing concentrations of complementary ssDNA, with capacitance increasing after hybridisation with increasing DNA target concentrations. The inset shows a control response for increasing concentrations of complete mismatch non-complementary ssDNA; the fluctuations are attributed to small amounts of non-specific interactions of the mismatch ssDNA sequence with the probe layer.

Below 10 kHz the behaviour between the two test devices was found to be unreliable. It is well reported that the low-frequency dielectric response is dominated by the ionic relaxation of the buffer [32,41,42,43], complicating detection. The complex composition of the PBS buffer (0.01 M phosphate buffer, 2.7 mM KCl, 0.137 M NaCl) may be the source of this behaviour. 

**Figure 11:**
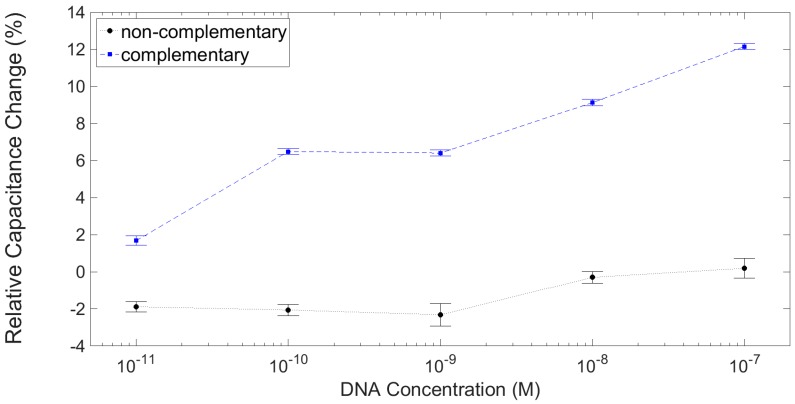
Dose response showing the relative change in capacitance from the ssPNA probe layer at 25 kHz for a device incubated with increasing concentrations of complementary ssDNA, and for a control device with increasing concentrations of complete mismatch non-complementary ssDNA. Error bars indicate ± σ, n = 5.

Above 10 kHz the response was much more reliable between the devices; a mid-frequency value of 25 kHz was chosen to calculate the relative percentage shifts in capacitance from the ssPNA probe layer. The response of a test and of a control are plotted in Figure 11. The second test device showed far lower sensitivity, with a percentage increase of <4% for 100 nM complementary ssDNA. The capacitance of the second control device dropped severely (~10%) between the probe layer measurement and ssDNA hybridisation, presumably due to mishandling. However, with increasing concentrations of mismatched ssDNA the capacitance deviated by no more than ~1%. 

Due to the lack of devices used for the experiment it is difficult to make any firm conclusions. One of the test devices showed little change in capacitance, with a maximum shift of <5% at 100 nM concentration, whereas the other test device showed a ~12% shift at the same concentration. Both of the control devices showed little change (±1%) with increasing concentrations of mismatched ssDNA sequences.

Regeneration of the probe layer by washing in 90 °C for 50 min with mechanical rocking was unsuccessful. Inspection of the devices showed some delamination of the SU-8 layers, which would cause increased electrostatic fringing fields and, therefore, no way to reproduce similar results. In the future the SU-8 passivation layer could be replaced with a self-aligned passivation layer: either Ta, anodised to form Ta_2_O_3_ or, alternatively, a SiN_x_ layer followed by a CHF_3_/O_2_ dry etch. Nonetheless, the dose response obtained for the device in Figure 11 shows the applicability of the sensors for direct capacitance detection of DNA hybridization.

## 4. Conclusions

We have shown two new methods to create large-area nanogap sensors in both horizontal and vertical coplanar configurations. For the horizontal coplanar device, the electron-beam lithography (EBL) steps used to pattern the liftoff layers could be replaced with a mechanical deformation nanoimprint lithography (NIL) method [44]. This would provide far greater fabrication throughput at the wafer level and scope to significantly reduce cost. Fabrication of the vertical coplanar device uses a reliable anisotropic dry etch process to control nanogap separation followed by anodic bonding to permanently bond the two sensor halves. Although the bonding parameters were optimised, there is likely to be a limit to the minimum interelectrode distance that can be achieved without causing collapse. Both the etching and bonding steps are easily performed at the wafer level, meaning that mass-production would be feasible. Using the horizontal coplanar nanogap device as an example, we showed that dielectric spectroscopy could be used as a method for the detection of PNA−DNA binding. 

Large area nanogap devices offer an exciting platform to explore direct capacitive/dielectric detection of biomolecules, as well as other direct electrochemical detection techniques, such as redox cycling signal amplification [29,45]. The fabrication processes presented for both vertical and horizontal coplanar, large-area, nanogap devices can be readily explored for the development of a wide range of geometries and (bio)sensing applications.

## Figures and Tables

**Table 1:** Variety of fabricated nanogaps suited towards electrochemical sensing applications.

Fabrication	Type	Orientation	Electrode	Gap	Area	Reference
EBL + SE (Cr)	Nanofluidic linear IDA	H	Pt	~250 nm	~1.4 × 10^−9^ m^2^	Goluch et al. [30]
ES	Parallel plate	H	Pt	~50 nm	~2.0 × 10^−12^ m^2^	Kim et al. [12]
FIB	Parallel plate	H	Au	~60 nm	~1.6 × 10^−13^ m^2^	Hatsuki et al. [31]
FIB	Parallel plate	H	Au	~510 nm	~2.5 × 10^−12^ m^2^	Hatsuki et al. [32]
EBL	Parallel plate	H	Au	~200 nm	~2.4 × 10^−12^ m^2^	Hsueh et al. [33]
EBL	Serpentine parallel plate	H	Au	~215 nm	~3.3 × 10^−9^ m^2^	This work
SE (SiO_2_)	Nanocavity linear array	V	Si	~90 nm	~2.1 × 10^−7^ m^2^	Ionescu-Zanetti et al. [34]
SE (SiO_2_) + AD	Nanocavity	V	Au	~65 nm	~2.0 × 10^−13^ m^2^	Strobel et al. [17]
SE (Cr)	Nanofluidic parallel plate cavity	V	Pt	~70 nm	~1.5 × 10^−10^ m^2^	Zevenbergen et al. [20]
SE (Cr)	Nanocavity crossbar array	V	Pt	~65 nm	~1.7 × 10^−12^ m^2^	Kätelhön et al. [27]
SE (Cr)	Nanofluidic parallel plate cavity	V	Au	~200 nm	~3.0 × 10^−11^ m^2^	Rassaei et al. [26]
EBL + SE (Si_3_N_4_)	Nanoporous ring-ring array	V	Pt	~100 nm	~3.6 × 10^−^^9^ m^2^	Hüske et al. [28]
SE (Cr)	Nanocavity ring-ring array	V	Pt	~230 nm	2.7 × 10^−^^7^ m^2^	Kanno et al. [35]
ICP RIE + AB	Parallel plate	V	Au	~500 nm	3.0 × 10^−^^6^ m^2^	This work

Key: AB = anodic bonding, AD = angled deposition, EBL = electron-beam lithography, ES = electrochemical synthesis, FIB = focussed ion beam, H= horizontal, ICP = inductively-coupled plasma, IDA = interdigitated array, RIE = reactive ion etching, SE = sacrificial etch and V = vertical.

**Table 2:** ICP-RIE anisotropic recipe used to etch the sub-micron channel in silicon.

Parameter	Value
SF_6_	50 SCCM
O_2_	8 SCCM
RF	90 W
ICP	8 s 1300 W ^1^, 32 s 1200 W
Pressure	10 mTorr
He backing	5 SCCM
Temperature	0.0 °C

^1^ stabilisation period.
